# Supplementary material for: PTEN/PKM2/ERα-Driven Glyoxalase 1 Overexpression Sustains PC3 Prostate Cancer Cell Growth Through MG-H1/RAGE Pathway Desensitization Leading to H2O2-Dependent KRIT1 Downregulation
Source: Antioxidants (Basel). 2025 Sep 15;14(9):1120. doi: 10.3390/antiox14091120 (PMC12466846; doi:10.3390/antiox14091120)
Supplement: Supplementary file 1 [file antioxidants-14-01120-s001.zip › antioxidants-3817685-(Figure S4)Original images and blots.pdf]

Figure a-b

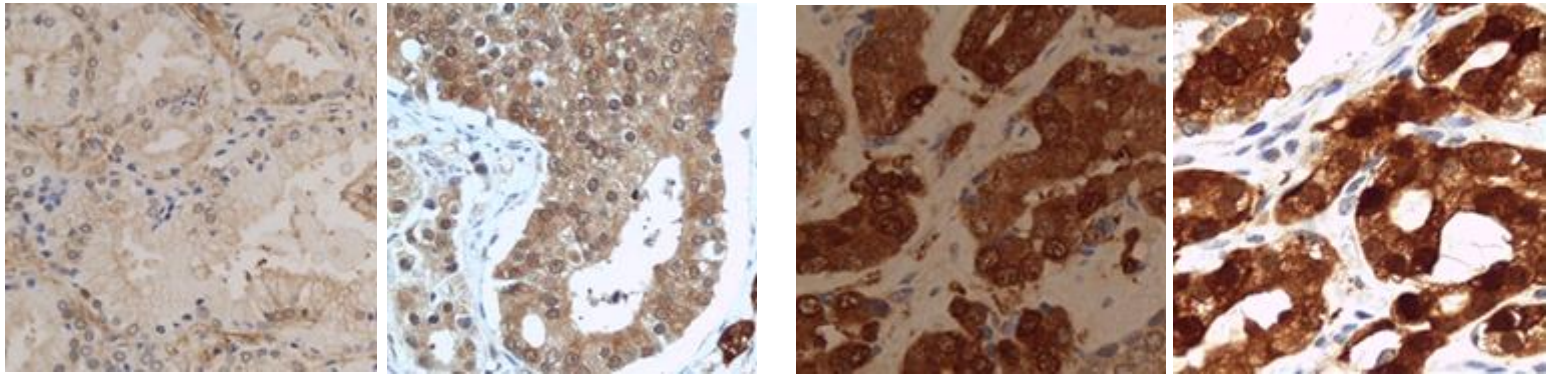

Figure 1e

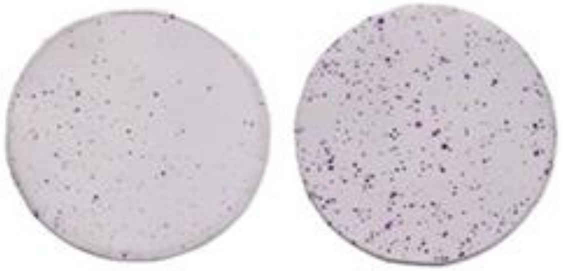

Full unedited gel for Figure 4a – PC3

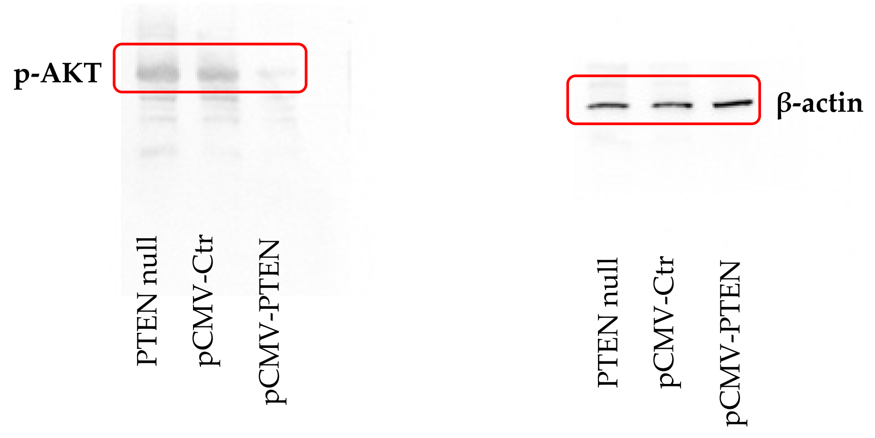

Full unedited gel for Figure 4b – DU145

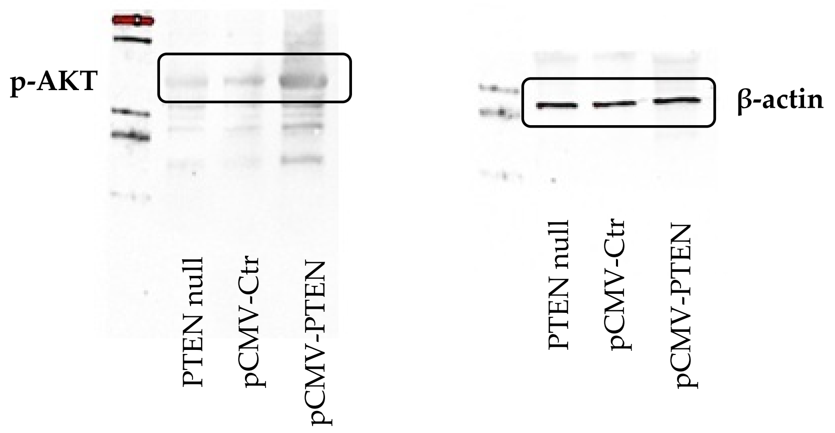

Full-size original blot for Figure 4a – PC3 and 4b – DU145

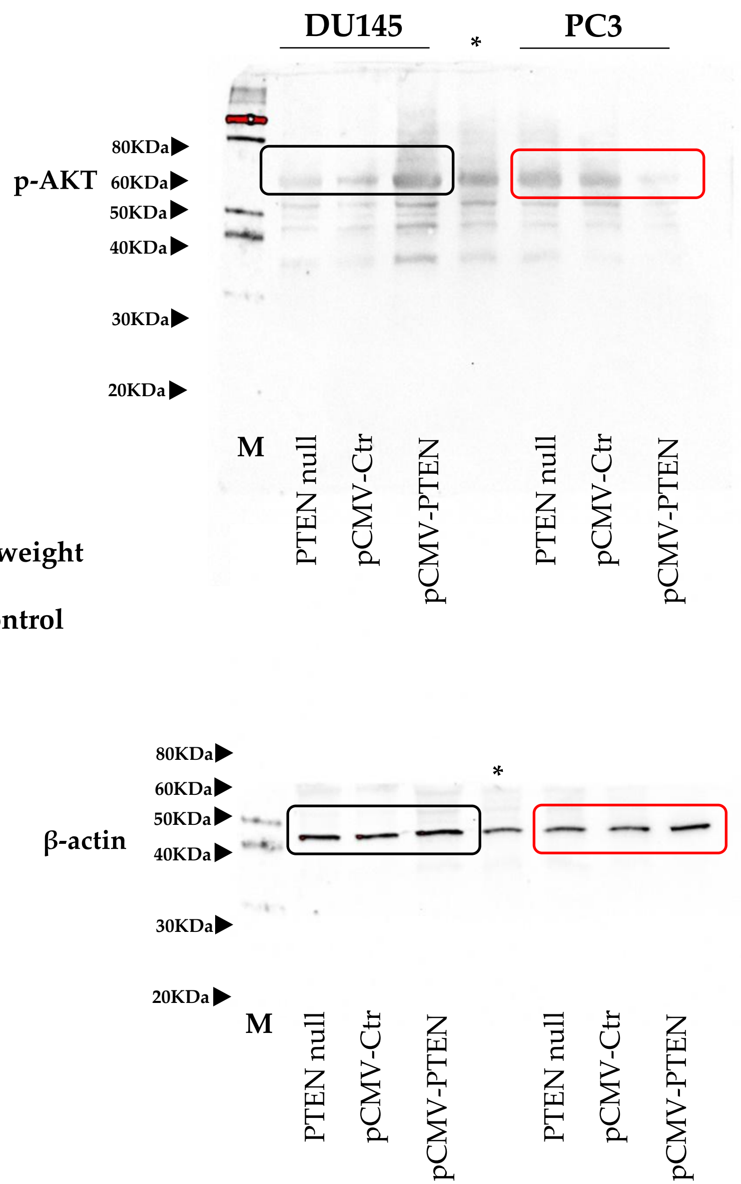

M: molecular weight marker
*: positive control

Full unedited gel for Figure 4d - DU145

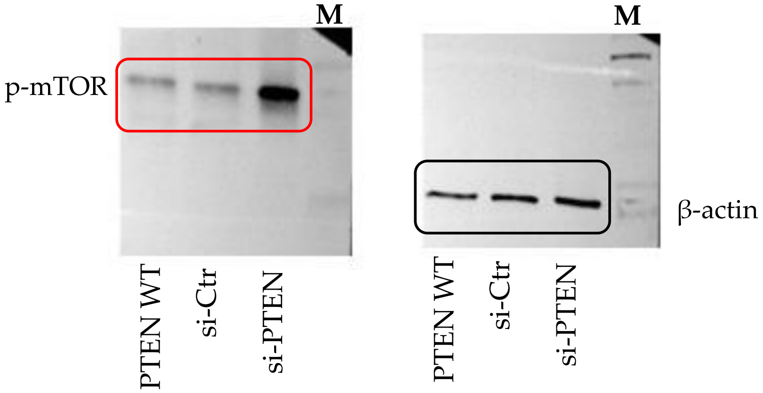

Full-size original blot for Figure 4d - DU145

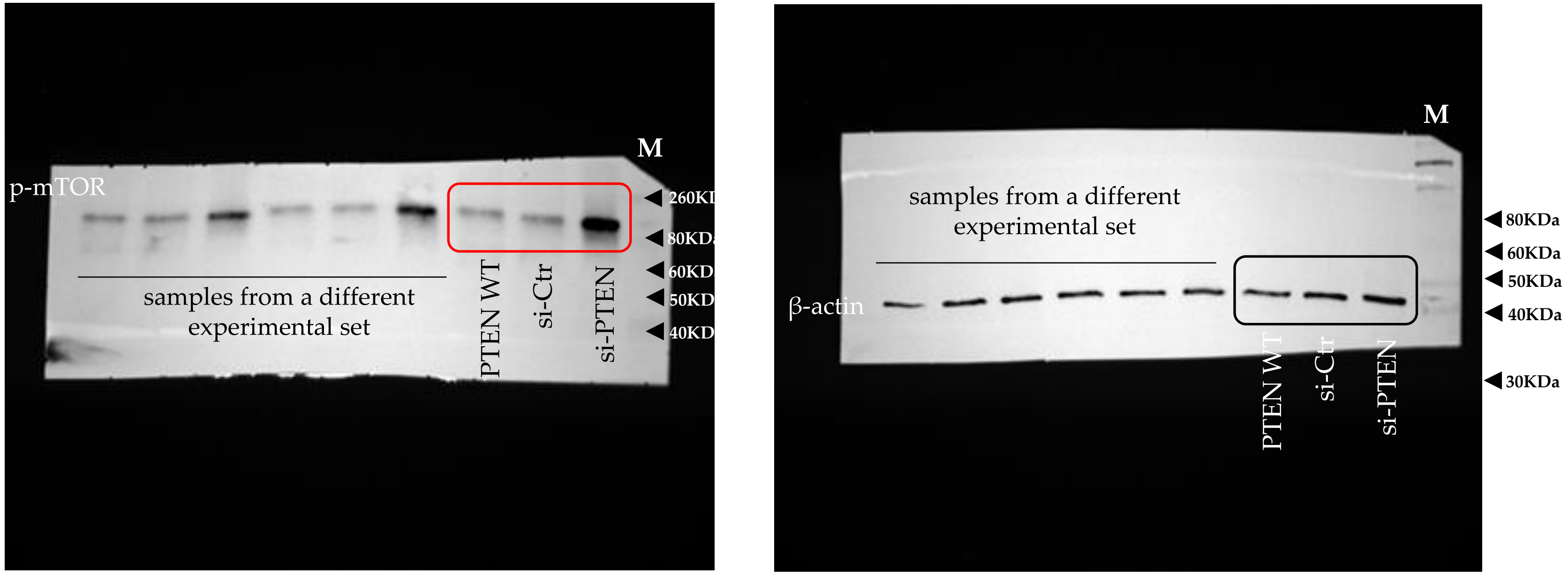

Full unedited gel for Figure 4c – PC3

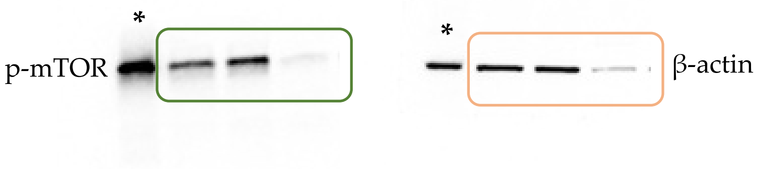

* Positive Control

Full-size original blot for Figure 4c – PC3

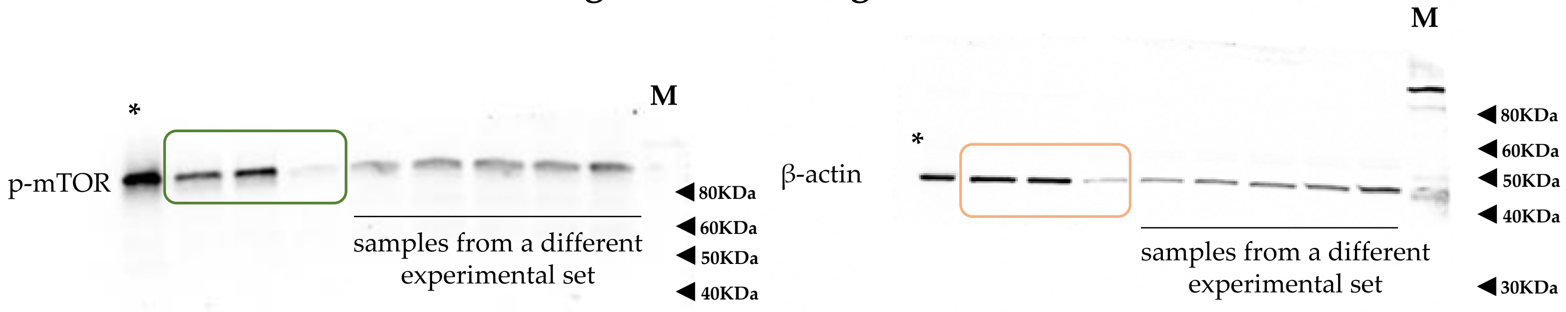

M: molecular weight marker

Full unedited gel/Full-size original blot for Figure 5a

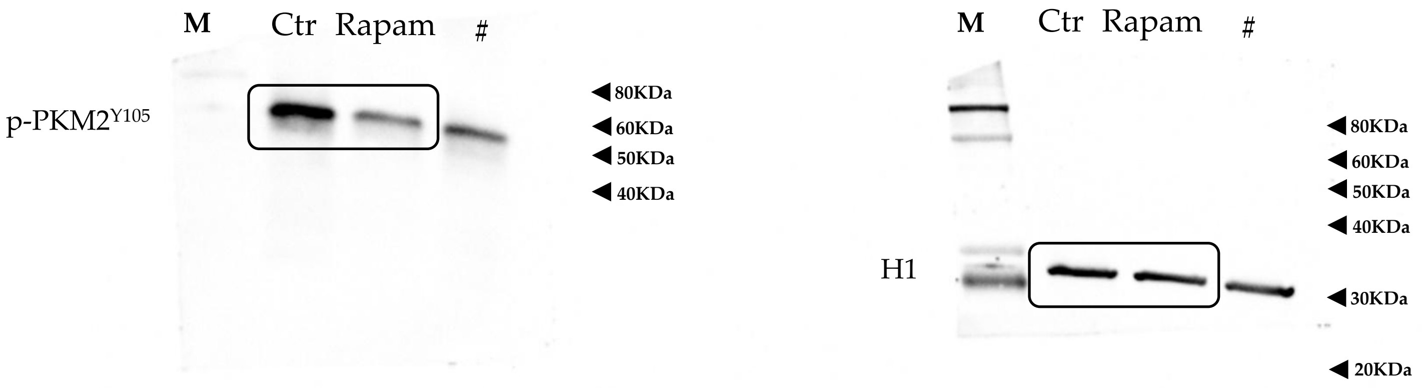

M: molecular weight marker

Positive Control

Full unedited gel/Full-size original blot for Figure 5b

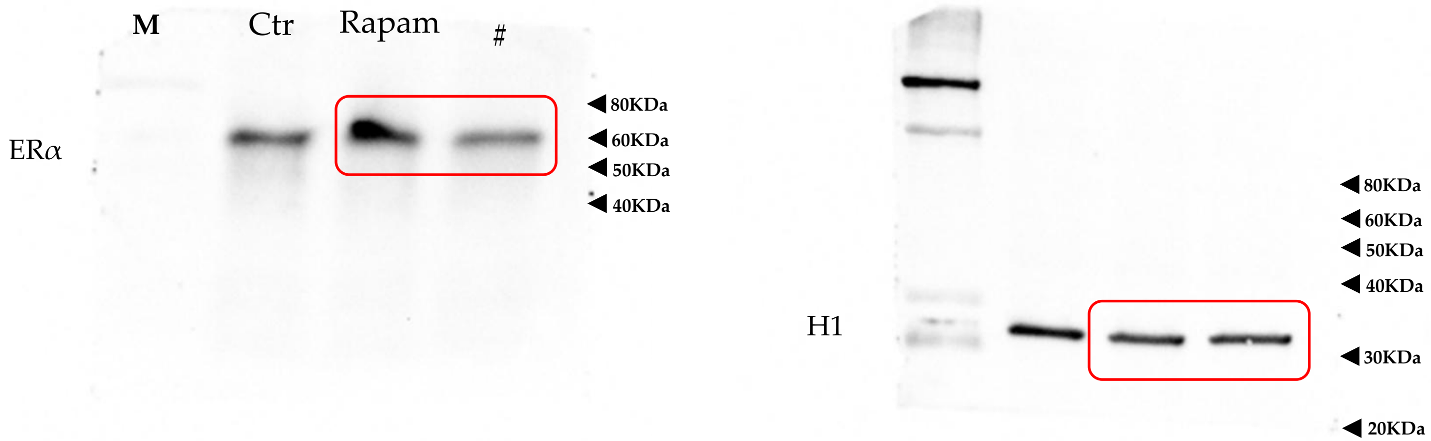

M: molecular weight marker

Positive Control

Full unedited gel/Full-size original blot for Figure 6a

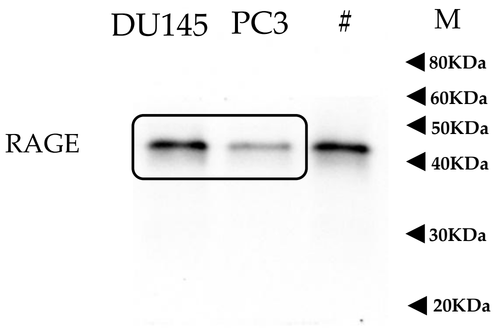

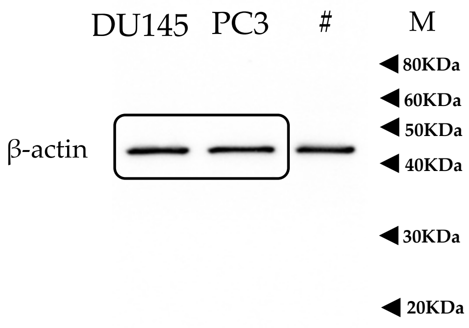

Positive Control

M: molecular weight marker
