# Supplementary material for: PTEN/PKM2/ERα-Driven Glyoxalase 1 Overexpression Sustains PC3 Prostate Cancer Cell Growth Through MG-H1/RAGE Pathway Desensitization Leading to H2O2-Dependent KRIT1 Downregulation
Source: Antioxidants (Basel). 2025 Sep 15;14(9):1120. doi: 10.3390/antiox14091120 (PMC12466846; doi:10.3390/antiox14091120)
Supplement: Supplementary file 1 [file antioxidants-14-01120-s001.zip › antioxidants-3817685-supplementary.pdf]

Supplementary Materials: PTEN/PKM2/ERα-driven glyoxalase 1 overexpression sustains PC3 prostate cancer cell growth through MG-H1/RAGE pathway desensitization leading to H_2O_2-dependent KRIT1 deregulation

Dominga Manfredelli, Camilla Torcoli, Marilena Pariano, Guido Bellezza, Tiziano Baroni, Vincenzo N. Talesa, Angelo Sidoni and Cinzia Antognelli

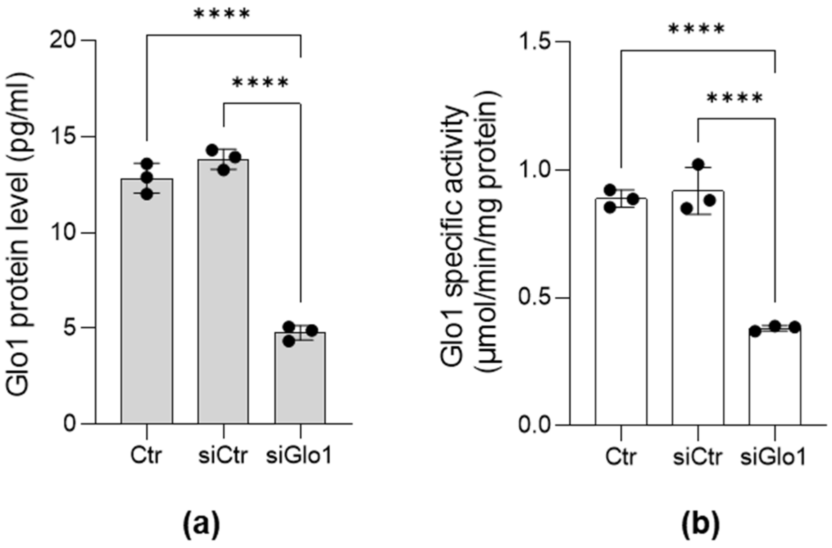

Figure S1 – Glyoxalase 1 (Glo1) silencing in PC3 cells. Glo1 smart-pool siRNA (siGlo1) efficiently suppresses **(a)** Glo1 protein levels, assessed by a specific ELISA kit, and **(b)** Glo1 specific enzyme activity, evaluated by the specific spectrophotometric assay, whereas it remained unaffected in control siRNA-transfected (siCtr) and untreated cells (Ctr). The histograms indicate means ± SD of three different cultures. ****$p < 0.0001$.

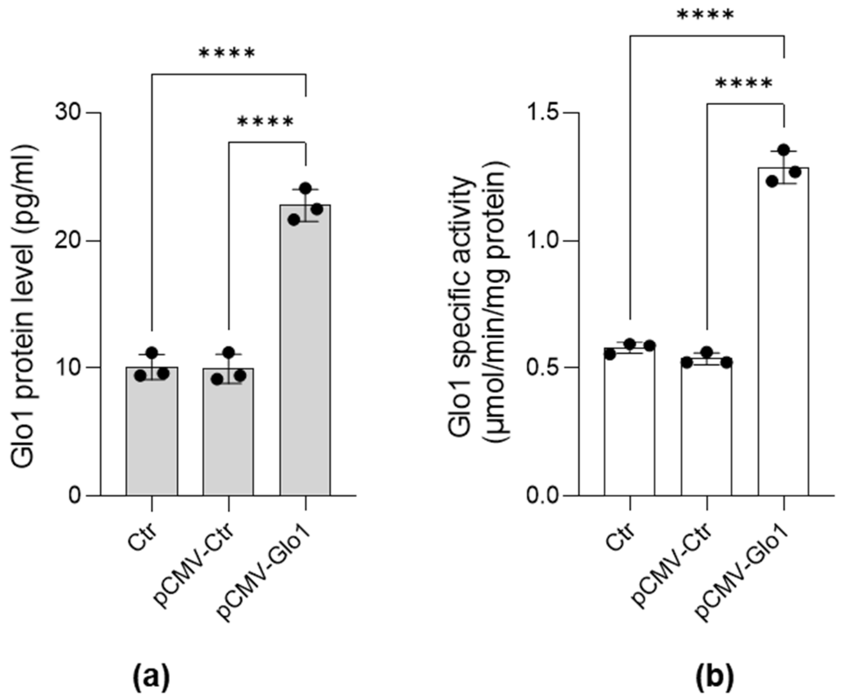

Figure S2 – Glyoxalase 1 (Glo1) overexpression in DU145 cells. Glo1 ectopic expression (pCMV-Glo1) efficiently induces **(a)** Glo1 protein levels, assessed by a specific ELISA kit, and **(b)** Glo1 specific enzyme activity, evaluated by the specific spectrophotometric assay, whereas it remained unaffected in control-transfected (pCMV-Ctr) and untreated cells (Ctr). The histograms indicate means ± SD of three different cultures. ****$p < 0.0001$.

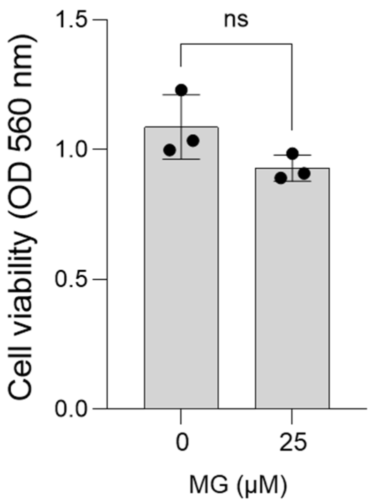

Figure S3 – Effect of methylglyoxal (MG) on PC3 cell viability. MG at the concentration of 25 µM was administrated to PC3 cells for 24 hours and cell viability was evaluated by Cell Counting Kit-8 (CCK-8) assay. The histograms indicate means ± SD of three different cultures. ns: not significant.
